# One-Year Outcome of Patients Undergoing Transcatheter Aortic Valve Replacement with Concomitant SignificantTricuspid Regurgitation

**DOI:** 10.3390/jcdd12050184

**Published:** 2025-05-14

**Authors:** Enrico Ferrari, Alberto Pozzoli, Catherine Klersy, Elena Caporali, Stefanos Demertzis, Giovanni Pedrazzini

**Affiliations:** 1 Cardiac Surgery Unit, Cardiocentro Ticino Institute, EOC, 6900 Lugano, Switzerland; 2 Biomedical Faculty, Università della Svizzera Italiana (USI), 6900 Lugano, Switzerland; 3 School of Medicine, University of Zurich, 8006 Zurich, Switzerland; 4 Clinical Epidemiology & Biostatistics, Fondazione IRCCS Policlinico San Matteo, 27100 Pavia, Italy; 5 Cardiology Unit, Cardiocentro Ticino Institute, EOC, 6900 Lugano, Switzerland

**Keywords:** transcatheter aortic valve replacement, tricuspid valve regurgitation, aortic valve stenosis

## Abstract

**Background**: The outcome of patients undergoing transcatheter aortic valve replacement (TAVR) can be affected by coexisting tricuspid regurgitation (TR). The aim of the study is to investigate the clinical results of patients undergoing TAVR with or without concomitant significant TR. **Methods**: Patients undergoing TAVR were divided into two groups according to TR severity: none/mild TR (low-grade) and moderate/severe TR (significant). Data were analysed and compared. Primary endpoint was the mortality 1-year. Secondary endpoints were re-hospitalization and the degree of postoperative and 1-year TR. **Results**: TAVR procedures were performed in 345 patients between September 2011 and February 2020. Median STS score was 4.3% (IQR: 2.6–7.2), median LVEF was 59.0% (IQR: 45.0–62.0), median aortic area was 0.70cm^2^ (IQR: 0.60–0.86), median mean gradient was 43.0mmHg (IQR: 36.0–53.0). Before TAVR, 297 patients (86.1%) had low-grade TR and 48 (13.9%) significant TR. Mean age was 82.4 ± 5.7 and 83.8 ± 6.2 years in low-grade and significant TR group, respectively (*p* = 0.109), with 47.5% (low-grade TR) and 56.3% (significant TR) of female patients (*p* = 0.279). Patients showed differences in EuroSCORE-II (3.2% (IQR: 1.9–5.7) in low-grade TR vs. 5.6% (IQR: 3.7–8.1) in significant TR; *p* < 0.001), impaired right ventricular function (3.0% vs. 20.8%; *p* < 0.001) and pulmonary hypertension (9.1% vs. 39.6%; *p* < 0.001). Mean valve size was 27.7 ± 2.9 mm. Hospital mortality was 2.0% in low-grade TR and 4.2% in significantTR patients (*p* = 0.308). Among discharged patients (*n* = 337), seven patients died within 30 days (2.0% low-grade TR; 2.1% significant TR; logrank test *p* = 0.154) and 40 were re-hospitalized for heart failure (11.1% low-grade TR; 14.6% significant TR; *p* = 0.470). After one year, 26 patients died, corresponding to a mortality of 7.9 deaths per 100-person year (95% CI 5.2–12.0) in low-grade TR group and 9.1 deaths per 100-person year (95% CI 3.4–24.3) in significant TR group (logrank test *p* = 0.815), with HR (low grade vs. significant TR) of 0.87, 95% CI 0.26–2.89. Re-hospitalization for heart failure was 16.5% and 19.6% for low-grade and significant TR, respectively (*p* = 0.713). Echocardiographic and functional changes over time showed no significant interaction between TR and time. **Conclusions**: In our experience, patients undergoing TAVR showed similar 30-day and 1-year outcome and re-hospitalization rate, regardless of the degree of concomitant tricuspid regurgitation.

## 1. Introduction

Transcatheter aortic valve replacement (TAVR) is a valid therapeutic option for patients at high-risk for surgery and for intermediate-risk patients suffering from severe symptomatic aortic valve stenosis. However, patients undergoing transcatheter procedures can suffer from multiple valve disease (approximately 20% of all TAVR population) and it is still not clearly understood whether a significant concomitant mitral (MR) or tricuspid regurgitation (TR) may affect the outcome of these patients. In particular, the significant tricuspid regurgitation can negatively affect the postoperative outcome of patients treated with TAVR, and some recent reports have investigated the role of this valve on mortality and rehospitalisation rate at short-term follow-up [1,2,3,4,5,6,7,8,9]. Published data suggest that after TAVR, patients with concomitant TR or MR can benefit of a slight improvement of the atrioventricular valve regurgitation on both sides, but some reports also suggest that the moderate/severe TR overload is independently associated with mortality, thus suggesting that the TR response to TAVR can be extremely variable [2,3]. Moreover, a persistent significant TR post-TAVR seems to be a major determinant of unfavourable outcome and therefore an early sequel intervention, such as a percutaneous tricuspid repair, should be considered [4,6]. Currently, the role of the significant TR is still under debate and can represent an important limitation to symptoms relief after TAVR, leading to repeated hospitalizations and death. The present study aims at investigating the hospital results and the 1-year outcome of patients undergoing TAVR with or without significant TR.

## 2. Methods

### 2.1. Study Design

This is a single centre longitudinal retrospective observational study including patients with severe symptomatic aortic valve stenosis undergoing TAVR through a trans-vascular access (the transapical TAVR were excluded given the possibility of mechanical interactions with the ventricular motion and the atrio-ventricular valve). Patients were divided into two groups according to the TR severity: none/mild TR (TAVR-low-grade TR group; the control group) and moderate/severe TR (TAVR-significant TR group; the study group). Data extracted from the institutional database and from the national Swiss TAVI-Registry database were analysed and compared between groups. Primary endpoint was the mortality at at 1 year. Secondary endpoints were the post-discharge re-hospitalization rate and the degree of TR at discharge and at 1-year follow-up. The present investigation abides the principles outlined in the Declaration of Helsinki (Ethical Principles for Medical Research Involving Human Subjects) adopted by the 18th WMA General Assembly in Helsinki, Finland, June 1964.

### 2.2. Patient Selection

Adult patients with severe symptomatic aortic valve stenosis, with moderate or high surgical risk profile, or patients aged above 80 were considered eligible for TAVR by the local Heart-Team. Before the procedure, all patients were under optimal medical treatment for cardiac decompensation and undergo transthoracic and/or transoesophageal echocardiography. The vascular access was investigated by means of angiographic computed tomography scan (CT-scan). Standard measurements of the left ventricular (LV) chamber dimensions, LV ejection fraction (LVEF), LV mass and left atrial volume were performed.

Specifically, moderate TR was evaluated quantitatively by the following metrics: an effective regurgitant orifice area of 0.2–0.39 cm^2^ (proximal isovelocity hemispheric surface area) or a regurgitant volume of 30–44 mL. The severe TR was evaluated quantitatively by the following metrics: an effective regurgitant orifice area ≥ 0.40 cm^2^ (proximal isovelocity hemispheric surface area) or a regurgitant volume ≥ 45 mL (2021 ESC/EACTS guidelines for valve disease). The presence of tricuspid regurgitation was categorized as low-grade or significant following standard echocardiographic criteria and guidelines. The right ventricular systolic pressure was evaluated using the TR jet peak velocity. Semi quantitative signs included hepatic vein systolic flow reversal, the inferior vena cava collapsibility index and vena contracta width ≥ 0.7 cm. Right ventricular (RV) ejection fraction (RVEF) ≤ 40% was adopted as a marker of RV systolic dysfunction following important findings of recent researches on magnetic resonance cardiac imaging [10]. Pulmonary artery systolic pressure (PASP) is often used as a surrogate measure of RV afterload and it was determined from the sum of the TR gradient calculated from peak transvalvular tricuspid velocity and semi-quantitatively estimated right atrial pressure. PASP ≥ 50 mmHg was adopted as marker of pulmonary hypertension following the standard definition of mild pulmonary hypertension (35–50 mm Hg), moderate pulmonary hypertension (50–70 mm Hg), and severe pulmonary hypertension (>70 mm Hg). Changes in the severity of TR were documented with echocardiographic controls at hospital discharge, one month after TAVR and after one year (planned echocardiographic and clinical examinations). The clinical and echocardiographic follow-up was 100% complete for all alive patients. All patients provided a signed informed consent for the TAVR procedure as well as an informed consent for the enrolment in the nationwide Swiss TAVI-Registry database (registered at clinicaltrials.gov, n. NCT01368250), approved by Ethic Committee (number 056/11).

### 2.3. TAVR Procedures

All procedures were performed in the hybrid room, under general anaesthesia and through a vascular access. Implanted valves were self-expanding (CoreValve (Medtronic Inc., Minneapolis, MN, USA), (Boston Scientific Corp., Maple Grove, MN, USA), PORTICO (Abbott Laboratories, Chicago, IL, USA), ACURATE (Boston Scientific Corp., Maple Grove, MN, USA)) or balloon expandable (SAPIEN valve (Edwards Lifesciences Inc., Irvine, CA, USA)). Prosthetic valve size was determined by means of preoperative three-dimensional CT-scan images (3-Mensio system (Pie Medical Imaging, Maastricht, Netherlands)). The primary access was the transfemoral followed by the direct transaortic, the trans-subclavian and the trans-carotid. All complications were collected and categorized following the VARC-3 definitions [11]

### 2.4. Statistical Analysis

We used the Stata software (ver.17, release 17, StatCorp, College Station, TX, USA) for all computations. We considered a 2-sided *p*-value < 0.05 as statistically significant. We described continuous variables with the mean and standard deviation (SD), or the median and 25th–75th percentiles when skewed. We compared them between groups with the Mann Whitney U test. We described categorical variables as counts and percent and compared them with the Fisher exact test. We used the logrank test to compare survival between patients with low-grade TR and with significant TR, and we plotted Kaplan Meier cumulative survival curves. We computed hazard ratios (HR) and 95% confidence intervals (95% CI) by means of a Cox model. Given the low number of deaths, we did not fit multivariable models. We compared changes over time (discharge, 30 days and 1 year) with generalized regression models and either identity or logistic link, depending on the response variable. To compare profiles over time by TR, we included an interaction term. We computed Huber-White robust standard errors to account for intra-subject correlation of measures.

## 3. Results

From September 2011 to February 2020, 345 patients with severe symptomatic aortic valve stenosis underwent a TAVR through a trans-vascular access. The majority (71%) were on New York Heart Association (NYHA) functional class II-III in the two weeks before the procedure. The median Society of Thoracic Surgeons (STS) risk score and interquartile range (IQR) was 4.3% (IQR: 2.6–7.2). Median LVEF was 59.0% (IQR: 45.0–62.0). The median aortic valve area (AVA) was 0.70cm^2^ (IQR: 0.60–0.86), the median peak valve gradient was 70.0mmHg (IQR: 56.0–84.0), and the median mean valve gradient was 43.0mmHg (IQR: 36.0–53.0).

Before TAVR, 297 patients (86.1%) presented a low-grade TR and 48 (13.9%) a significant TR (13 severe TR and 35 moderate TR as per definition). Pre-operative unadjusted characteristics and baseline echocardiographic findings of the entire group and subgroups are shown in Table 1. Mean age was 82.4 ± 5.7 and 83.8 ± 6.2 years in the low-grade TR and significant TR group, respectively (*p* = 0.109), while the female gender accounted for 47.5% and 56.3%, respectively (*p* = 0.279). Patients showed significant differences in pre-operative risk scores: EuroSCORE-II was 3.2% (IQR: 1.9–5.7) for the low-grade TR group and 5.6% (IQR: 3.7–8.1) for the significant TR group (*p* < 0.001); STS-score was 4.1% (IQR: 2.5–7.1) for low-grade TR group and 5.4% (IQR: 3.4–8.5) for the significant TR group (*p* = 0.011). Median AVA was 0.72cm^2^ (IQR: 0.60–0.89) in the group of patients with low-grade TR and 0.64cm^2^ (IQR: 0.51–0.70) for patients with significant TR (*p* = 0.002). Differences between subgroups in the incidence of impaired right ventricular function (3.0% vs. 20.8%; *p* < 0.001) and pulmonary hypertension (9.1% vs. 39.6%; *p* < 0.001) were detected.

Procedural details are summarized in Table 2. The mean prosthetic valve size was 27.7 ± 2.9mm. As far as the type of the valve is concerned, 213 patients (61.8%) received a CoreValve, 86 patients (24.9%) a SAPIEN, 25 patients (7.2%) an ACURATE, 17 patients (4.9%) received a Lotus valve and 4 patients (1.2%) received a PORTICO. The trans-carotid access was used in two patients (0.6%), the trans-subclavian in three (0.9%), and the direct transaortic in 36 (10.4%). All other cases were transfemoral (88.1%). There were no differences between groups concerning the procedural time and the amount of contrast injected.

### 3.1. Hospital Results and 30-Day Outcome

**Figure 1:**
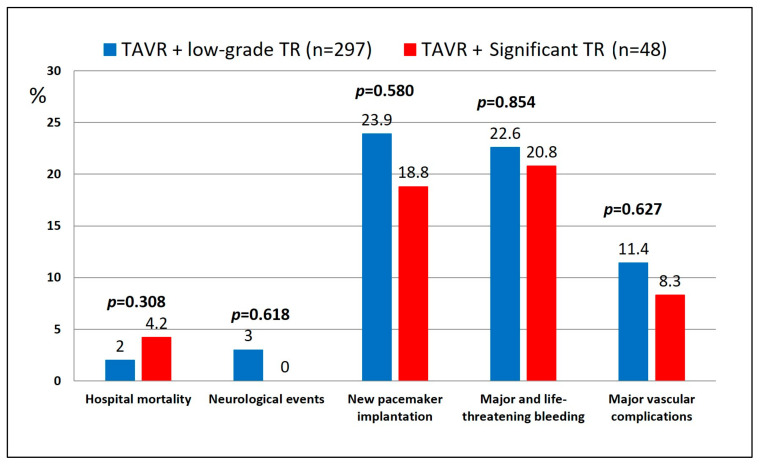
Postoperative complications after TAVR in patients with and without significant tricuspid regurgitation (according to VARC-3 definitions).

Eight patients died in hospital, 6 (2.0%) among the low-grade TR group and 2 (4.2%) in the significant TR group (*p* = 0.308) (Table 3) (Figure 1). Major vascular complications occurred in 11.0% of cases (*p* = 0.0627) and a new pacemaker (PM) was implanted in 80 patients (23.2%) (23.9% in low-grade TR cases; 18.8% in significant TR cases; *p* = 0.580). Among all discharged patients (n = 337), 7 patients died within 30 days: 6 (2.0%) in the low-grade TR group and 1 (2.1%) in the significant TR group (logrank test *p* = 0.154). Forty patients (11.1% in low-grade TR and 14.6% in significant TR; *p* = 0.470) were re-hospitalized for symptoms of worsening heart failure within 30 days.

### 3.2. One-Year Outcome

**Figure 2:**
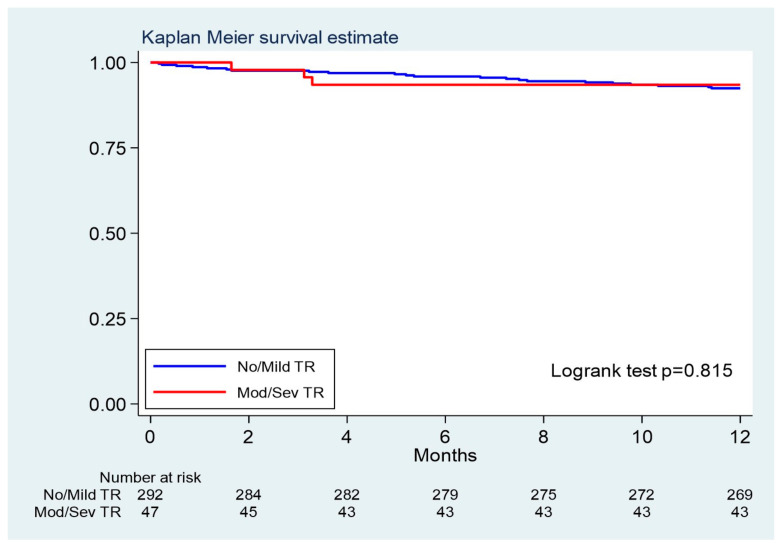
Kaplan–Meier curve showing the survival at 1 year for patients undergoing TAVR with and without significant tricuspid regurgitation.

Among all discharged patients (n = 337), 26 died within 1 year (including 7 cases who died within 30 days), corresponding to a mortality of 7.9 deaths per 100-person year (95%CI 5.2–12.0, 22 patients) in the low-grade TR group and 9.1 deaths per 100-person year (95%CI 3.4–24.3, 4 patients) in the significant TR group (logrank test *p* = 0.815), with an HR (low grade vs. significant TR) of 0.87, 95% CI 0.26–2.89 (Table 3) (Figure 2). Re-hospitalization for heart failure during the follow-up occurred in 16.5% and 19.6% of patients with low-grade and significant TR, respectively (*p* = 0.713).

### 3.3. Functional and Echocardiographic Changes over Time

Echocardiographic and NYHA class changes over time are shown in Table 4. Considering all patients, peak and mean gradients at discharge were 15.6 ± 7.4 mmHg and 8.4 ± 4.1 mmHg, respectively. Significant changes were identified while comparing preoperative versus 1-year echocardiographic data, with improved LVEF (*p* < 0.001), LV mass (*p* = 0.008) and pulmonary hypertension (*p* < 0.001). NYHA functional class III-IV also improved after TAVR (*p* < 0.001). Concerning the tricuspid regurgitation, we did not detect a significant interaction between the degree of the TR and the time, showing that the profile over time is not different for none/mild vs. mod/severe TR; for these reason only changes over the entire case series are presented in Table 4, while Table 5 shows the same data for each TR group.

## 4. Discussion

In order to improve the clinical outcome and prevent re-hospitalization, patients undergoing surgical AVR can also be subjected, if needed, to concomitant left or right atrio-ventricular valve repair or replacement during the same surgical session. On the other hand, TAVR is still performed as a stand-alone procedure and patients with a concomitant severe tricuspid regurgitation traditionally cannot undergo, at the same time, a double valve treatment. Moreover, not all centres performing TAVR can offer transcatheter tricuspid procedures, yet. Therefore, patients with a significant TR remain patients at risk of worse clinical outcome and repeated re-hospitalizations even after TAVR when compared to patients with low-grade TR, but this is yet to be confirmed and the topic is still under debate. In order to verify the weight of the untreated significant TR on clinical outcome at short-term follow-up, we analysed and compared data from patients undergoing TAVR with or without concomitant significant TR.

The most important finding of the study is that all patients had similar 30-day and 1-year clinical outcome regardless the severity of the preoperative concomitant tricuspid regurgitation. Moreover, in our cohort the number of patients with significant TR showed a positive trend over time and after TAVR the number of patients with significant TR improved from 13.9% at baseline to 11.5% at 30-day and to 9.3% at 1-year (despite data did not reached statistical significance). Clinically, this has an impact with symptoms and while the 79% of patients in the significant TR group suffered from NYHA class III-IV before TAVR, only 9% showed the same symptoms at 1-year follow-up (Table 5).

These findings are encouraging considering that, preoperatively, patients included in the two groups showed important clinical and echocardiographic differences. In particular, patients included in the group with significant TR showed worst preoperative NYHA functional class, worst surgical risk profiles, impaired right ventricular function and higher rate of pulmonary hypertension, when compared to patients with low-grade TR. Traditionally, surgical patients with concomitant untreated significant TR with impaired right ventricular function have a higher risk of postoperative morbidity and mortality, as well as a higher risk of repeated rehospitalisation. Similar clinical consequences have also been found in papers reporting patients undergoing TAVR with concomitant significant TR [12,13,14,15,16,17,18,19]. In particular, a systematic review and meta-analysis published by Prasitlumkum described a cohort of 6255 patients with significant TR and 21,359 patients without significant TR (with proportion of TAVR patients with significant tricuspid regurgitation ranging from 7.44% to 42.7%, following the different sources). They concluded that the mortality increased by up to two-fold among patients undergoing TAVR with significant TR, suggesting that the severity of the TR should be considered an important component of a risk stratification tool [12]. Importantly, the authors also suggested that the presence of isolated significant tricuspid regurgitation (functional) could signify that these patients had more primary causes for tricuspid regurgitation, which may portend severe pathophysiology requiring a more aggressive surgical (rare) or interventional TR management to avoid poor outcomes. In this scenario, a more specific TR and RV evaluation during the preoperative assessment in TAVR patients can be of utmost importance for identifying patients that present a higher risk profile. Some studies also demonstrated that RV size could be considered one of the independent predictors of TAVR outcome. RV dilation reflects chronic and severe pressure and volume overload thus can be considered a sign of advanced RV dysfunction following severe TR.

Fan et al. also reported similar results in a systematic review including 6466 patients: the presence of a moderate or severe TR at baseline increased the all-cause mortality after TAVR and therefore the author advised that a careful assessment of the right ventricular function and valve should be carried out by the hospital Heart-Teams [13].

Compared to these results, our data diverge and though hospital mortality somehow differed between groups, yet not reaching statistical significance (2% vs. 4.2%; *p* = 0.069), we observed a similar mortality rate between the two groups after one year (7.6% vs. 8.7%; *p* = 0.815). Similarly, the re-hospitalization rate at 1-year was also comparable between the groups (16.5% vs. 19.6%; *p* = 0.713), suggesting that there might be a positive effect of the TAVR procedure on TR degree and, consequently, on short-term clinical outcome. This is also confirmed by another group that showed that, when adjusted to multiple echocardiographic characteristics, the baseline significant TR was not associated with long-term mortality following TAVR, while the persistent right dysfunction was associated with the highest mortality risk [20]. This can be explained by the fact that patients with severe AS, baseline significant TR and severe RV dysfunction may not have significant relief of post-capillary pulmonary hypertension after TAVR. What is more, the improved stroke volume after TAVR increases the systemic venous return, which could accelerate the dilation and failure of the right heart when combined with pulmonary hypertension. In our study, the group with a significant TR included 20% of patients with severe RV dysfunction. However, given the fact that in case of severe TR the RV function can be overestimated, patients with impaired right ventricle in this group were probably the majority and, in this view, the good clinical results at 1-year follow-up are even more important.

The selection criteria for TAVR should therefore also include a specific analysis of the right ventricular function, in order to better predict the outcomes. Magnetic Resonance cardiac imaging of the RV can also be useful for these purposes. Once the TAVR is performed, the changes in the degree of the TR as well as in right ventricular function should be used to rapidly act, surgically or percutaneously, in case of worsening of the hemodynamic status with consequent repeated hospitalizations for symptoms of heart failure. Following recent reports, the non-improved TR is associated with worst outcomes at mid-term follow-up and patients can be considered for further transcatheter tricuspid valve treatments such as the tricuspid edge-to-edge procedure, the placement of a percutaneous tricuspid annular ring or the implantation of a transcatheter tricuspid biological valve [21,22]. Therefore, the indication for the transcatheter tricuspid treatment after TAVR can be the presence of signs of worsening congestive heart failure and worsening RV function.

### Study Limitations

The present study has some limitations. This is a single centre retrospective study without randomization and with a follow-up limited to one year. Moreover, the influence related to the choice of the valve type or the access site, as well as the influence of specific tissue valves employed for the procedures were not included in the present analysis. There could also be a risk of bias because we do not know how many patients with severe AS and severe right ventricular dysfunction with significant TR were not considered eligible for a TAVR and therefore treated medically. Moreover, the follow-up of 1 year is short and the conclusions should be confirmed by means of a longer follow-up.

## 5. Conclusions

In our experience, patients undergoing TAVR have similar 30-day and 1-year outcome and re-hospitalization rate regardless the degree of the concomitant tricuspid regurgitation at time of TAVR, and showed a trend towards TR improvement at 1-year. Therefore, despite a longer follow-up is certainly required to confirm these findings, nowadays a concomitant moderate-to-severe TR should no longer be considered a limiting factor during the screening process for TAVR.

## Figures and Tables

**Table 1:** Patient characteristics and baseline echocardiographic findings.

	Overall (*n* = 345)	TAVR-Low-Grade TR (*n* = 297)	TAVR-Significant TR (*n* = 48)	*p*-Value
Age (years)	82.6 ± 5.8	82.4 ± 5.7	83.8 ± 6.2	0.109
Female gender	168 (48.7)	141 (47.5)	27 (56.3)	0.279
Body mass index (kg/m^2^)	26.5 ± 4.8	26.8 ± 4.8	24.6 ± 4.0	0.003
Hypertension	270 (78.3)	233 (78.5)	37 (77.1)	0.851
Diabetes	100 (29.0)	85 (28.6)	15 (31.3)	0.733
Chronic kidney failure	15 (4.3)	14 (4.7)	1 (2.1)	0.704
Dialysis	11 (3.2)	10 (3.4)	1 (2.1)	1.000
COPD	48 (13.9)	42 (14.1)	6 (12.5)	1.000
Cerebrovascular accident	34 (9.9)	28 (9.4)	6 (12.5)	0.446
Peripheral artery disease	106 (30.7)	89 (30.0)	17 (35.4)	0.500
NYHA III–IV	245 (71.0)	207 (69.7)	38 (79.2)	0.230
NYHA class				0.644
I	25 (7.3)	23 (7.7)	2 (4.2)	-
II	75 (21.7)	67 (22.6)	8 (16.7)	-
III	195 (56.5)	164 (55.2)	31 (64.6)	-
IV	50 (14.5)	43 (14.5)	7 (14.6)	-
Coronary artery disease	221 (64.0)	193 (65.0)	28 (58.3)	0.418
Myocardial infarction	53 (15.4)	42 (14.1)	11 (22.9)	0.131
Previous PCI	118 (34.2)	104 (35.0)	14 (29.2)	0.513
Atrial fibrillation	102 (29.6)	76 (25.6)	26 (54.2)	<0.001
Previous cardiac surgery	49 (14.2)	36 (12.1)	13 (27.1)	0.012
Previous pacemaker	43 (12.5)	31 (10.4)	12 (25.0)	0.009
Log. EuroSCORE-I (%)	11.25 (IQR: 6.6–18.3)	10.3 (IQR: 6.2–16.9)	16.6 (IQR: 11.7–28.9)	<0.001
EuroSCORE-II (%)	3.4 (IQR: 2.1–6.2)	3.2 (IQR: 1.9–5.7)	5.6 (IQR: 3.7–8.1)	<0.001
STS-score (%)	4.3 (IQR: 2.6–7.2)	4.1 (IQR: 2.5–7.1)	5.4 (IQR: 3.4–8.5)	0.011
Echocardiographic data				
LVEF (%)	59.0 (IQR: 45.0–62.0)	60.0 (IQR: 47.0–62.0)	55.0 (IQR: 38.0–60.0)	0.064
Aortic peak gradient (mmHg)	70.0 (IQR: 56.0–84.0)	70.0 (IQR: 59.0–85.0)	66.0 (IQR: 45.0–81.0)	0.159
Aortic mean gradient (mmHg)	43.0 (IQR: 36.0–53.0)	43.0 (IQR: 37.0–53.0)	42.0 (IQR: 28.0–52.0)	0.279
Aortic valve area (cm^2^)	0.70 (IQR: 0.60–0.86)	0.72 (IQR: 0.60–0.89)	0.64 (IQR: 0.51–0.70)	0.002
LV mass (g)	216.0 (IQR: 170.0–254.0)	217.0 (IQR: 167.0–250.0)	208.0 (IQR: 174.0–267.0)	0.658
Indexed LV mass (g/m^2^)	121.0 (IQR: 102.0–142.0)	120.0 (IQR: 100.5–241.0)	129.0 (IQR: 117.0–151.0)	0.060
Impaired RV function (RVEF < 40%)	19 (5.5)	9 (3.0)	10 (20.8)	<0.001
Pulmonary hypertension (PASP ≥ 50 mmHg)	46 (13.3)	27 (9.1)	19 (39.6)	<0.001

Values are expressed as mean ± standard deviation or median (IQR: 25th-75th percentiles) if continuous or n (%) if categorical. TAVR: transcatheter aortic valve replacement; TR: tricuspid regurgitation; COPD: chronic obstructive pulmonary disease; EuroSCORE: European System for Cardiac Operative Risk Evaluation; NYHA: New York Heart Association; PCI: percutaneous coronary intervention; STS: Society of Thoracic Surgeons; LVEF: left ventricular ejection fraction; LV: left ventricle; RV: right ventricle; PASP: Pulmonary artery systolic pressure.

**Table 2:** Procedural details.

	Overall (*n* = 345)	TAVR-Low-Grade TR (*n* = 297)	TAVR-Significant TR (*n* = 48)	*p*-Value
Mean TAVR valve size (mm)	27.7 ± 2.9	27.7 ± 3.0	27.1 ± 2.3	0.219
LOTUS valve	17 (4.9)	15 (5.1)	2 (4.2)	-
Edwards SAPIEN	2 (0.6)	1 (0.3)	1 (2.1)	-
Edwards SAPIEN-XT	6 (1.7)	6 (2.0)	0	-
Edwards SAPIEN-3	75 (21.7)	63 (21.2)	12 (25.0)	-
Edwards SAPIEN-3 ULTRA	3 (0.9)	2 (0.7)	1 (2.1)	-
Medtronic CoreValve	70 (20.3)	57 (19.2)	13 (27.1)	-
Medtronic CoreValve Evolut PRO	2 (0.6)	1 (0.3)	1 (2.1)	-
Medtronic CoreValve Evolut R	141 (40.9)	127 (42.8)	14 (29.3)	-
St. Jude Medical PORTICO	4 (1.2)	3 (1.0)	1 (2.1)	-
Boston Scientific ACURATE	25 (7.2)	22 (7.4)	3 (6.3)	-
Vascular access: - Transfemoral - Transaortic - Trans-subclavian - Trans-carotid	304 (88.1) 36 (10.4) 3 (0.9) 2 (0.6)	262 (88.2) 30 (10.1) 3 (1.0) 2 (0.7)	42 (87.5) 6 (12.5) 0 0	0.914
Use of vascular closure device	265 (76.8)	227 (76.4)	38 (79.2)	0.309
Use of embolic protection device	13 (3.8)	10 (3.4)	3 (6.3)	0.403
Balloon diameter for BAV (*n* = 338) (mm)	21.6 ± 2.2	21.6 ± 2.2	21.4 ± 2.3	0.655
Contrast (mL)	285.3 ± 132.1	288.0 ± 134.7	268.4 ± 114.2	0.423
Procedural time (min)	104.4 ± 46.3	104.8 ± 47.0	102.4 ± 41.3	0.709

Values are expressed as mean ± standard deviation or median (25th–75th percentiles) if continuous or n (%) if categorical. TAVR: transcatheter aortic valve replacement; TR: tricuspid regurgitation; BAV: balloon aortic valvuloplasty.

**Table 3:** Outcomes and complications.

*30-Day Outcomes*	Overall (*n* = 345)	TAVR-Low-Grade TR (*n* = 297)	TAVR-Significant TR (*n* = 48)	*p*-Value
Valve dislocation/embolisation	5 (1.4)	4 (1.3)	1 (2.1)	0.529
Major vascular complications	38 (11.0)	34 (11.4)	4 (8.3)	0.627
Major and life-threatening bleeding	77 (22.3)	67 (22.6)	10 (20.8)	0.854
Median red blood transfusions (bags)	0 (IQR: 0.0–2.0)	0 (IQR:0.0–2.0)	0 (IQR: 0.0–1.0)	0.555
New PCI (coronary occlusion)	1 (0.3)	1 (0.3)	0	1.000
New pacemaker implantation	80 (23.2)	71 (23.9)	9 (18.8)	0.580
Acute kidney injury	15 (4.3)	13 (4.4)	2 (4.2)	1.000
Neurological events	9 (2.6)	9 (3.0)	0	0.618
New onset of atrial fibrillation	32 (12.7)	20 (9.3)	12 (32.4)	<0.001
ICU stay (days)	1.0 (IQR: 1.0–3.0)	1.0 (IQR: 1.0–2.0)	1.5 (IQR: 1.0–3.0)	0.436
Hospital stay (days)	6.0 (IQR: 4.0–8.0)	6.0 (IQR: 4.0–8.0)	6.0 (IQR: 4.0–8.0)	0.659
NYHA III–IV	12 (3.5)	8 (2.7)	4 (8.3)	0.069
**In-hospital mortality** - Multiple organ failure - Myocardial infarction - Cardiac tamponade - Cardiogenic shock - Haemoptysis for gastric cancer	8 (2.3) 2 (0.6) 1 (0.3) 2 (0.6) 2 (0.6) 1 (0.3)	6 (2.0) 0 1 (0.3) 2 (0.7) 2 (0.7) 1 (0.3)	2 (4.2) 2 (4.2) 0 0 0 0	0.308
**30-day mortality** (including in hospital) - Multiple organ failure - Myocardial infarction - Cardiac tamponade - Cardiogenic shock - Sudden death - Respiratory failure - Aortic root rupture - Haemoptysis for gastric cancer	15 (4.3) 3 (0.9) 2 (0.6) 2 (0.6) 4 (1.2) 1 (0.3) 1 (0.3) 1 (0.3) 1 (0.3)	12 (4.0) 0 2 (0.7) 2 (0.7) 4 (1.4) 1 (0.3) 1 (0.3) 1 (0.3) 1 (0.3)	3 (6.3) 3 (6.3) 0 0 0 0 0 0 0	0.070
Re-hospitalisation for heart failure	40 (11.6)	33 (11.1)	7 (14.6)	0.470
***1-year outcomes***	**Overall** **(*n* = 337)**	**TAVR-low-grade TR** **(*n* = 291)**	**TAVR-Significant TR** **(*n* = 46)**	***p*** **-value**
New PCI	2 (0.6)	2 (0.7)	0	0.734
New pacemaker implantation	2 (0.6)	2 (0.7)	0	1.000
Re-hospitalisation for heart failure	57 (16.9)	48 (16.5)	9 (19.6)	0.713
NYHA Class				0.331
NYHA Class I	91 (27.0)	79 (27.1)	12 (26.1)	-
NYHA Class II	143 (42.4)	124 (42.6)	19 (41.3)	-
NYHA Class III	9 (2.7)	6 (2.1)	3 (6.5)	-
NYHA Class IV	1 (0.3)	1 (0.3)	0	-
1-year mortality * **Number of deaths** Rate per 100 person year (95% CI) - Malignancy - Sepsis - Multiple organ failure - Myocardial infarction - Cardiogenic shock - Pulmonary embolism - Pneumonia - Cardiac arrest - Haemorrhagic stroke - Respiratory failure - Aortic root rupture - Sudden death - Unknown	26 (7.7) 8.1 (5.5–11.8) 5 (1.5) 3 (0.9) 2 (0.6) 2 (0.6) 2 (0.6) 2 (0.6) 2 (0.6) 1 (0.3) 1 (0.3) 1 (0.3) 1 (0.3) 1 (0.3) 3 (0.9)	22 (7.6) 7.9 (5.2–12.0) 5 (1.7) 3 (1.0) 0 2 (0.7) 2 (0.7) 2 (0.7) 1 (0.4) 0 1 (0.3) 1 (0.3) 1 (0.3) 1 (0.3) 3 (1.0)	4 (8.7) 9.1 (3.4–24.3) 0 0 2 (4.3) 0 0 0 1 (2.2) 1 (2.2) 0 0 0 0 0	0.815 **

Values are expressed as mean ± standard deviation or median (25th–75th percentiles) if continuous or n (%) if categorical. TAVR: transcatheter aortic valve replacement; ICU: intensive care unit; PCI: percutaneous coronary intervention; TR: tricuspid regurgitation; NYHA: New York Heart Association. * Including 30-day mortality, excluding in-hospital mortality. ** logrank test.

**Table 4:** Echocardiographic findings at different time points.

					Change over Time	Post Hoc Comparisons			Difference Between TR Time Profiles
	Preoperative (*n* = 345)	Discharge (*n* = 337)	30 Days (*n* = 330)	1-Year Follow-Up (*n* = 311)	Overall *p*-Value #	Discharge vs. Preoperative	30 Days vs. Discharge	1 Year vs. 30 Days	* *p* for Interaction
LVEF (%)	53.4 ± 12.6	54.2 ± 11.5	55.2 ± 10.6	55.6 ± 9.7	<0.001	*0.090*	*0.004*	*0.467*	0.482
Aortic peak gradient (mmHg)	71.4 ± 23.5	15.6 ± 7.4	16.2 ± 8.7	16.5 ± 11.2	<0.001	*<0.001*	*0.231*	*0.724*	0.836
Aortic mean gradient (mmHg)	44.7 ± 15.4	8.4 ± 4.1	8.8 ± 4.9	9.2 ± 7.0	<0.001	*<0.001*	*0.154*	*0.421*	0.925
Aortic orifice area (cm^2^)	0.7 ± 0.2	N.A.	N.A.	N.A.					
LV mass (g)	216.7 ± 72.3	210.4 ± 63.0	204.5 ± 67.6	195.0 ± 61.5	0.008	*0.133*	*0.264*	*0.200*	0.389
Indexed LV mass (g/m^2^)	123.8 ± 34.5	122.8 ± 42.5	115.8 ± 35.3	110.7 ± 31.4	<0.001	*0.678*	*0.011*	*0.181*	0.392
LVEDD (mm)	47.0 ± 8.0	46.8 ± 7.9	47.8 ± 8.3	46.6 ± 7.8	0.352	*0.719*	*0.097*	*0.118*	0.435
LVESD (mm)	33.8 ± 14.9	32.5 ± 15.2	32.1 ± 7.6	31.7 ± 8.0	0.046	*0.023*	*0.672*	*0.521*	0.176
Impaired RV function (RVEF < 40%)	19 (5.5)	17 (5.0)	6 (1.8)	3 (0.9)	0.082	*0.744*	*0.078*	*0.342*	0.407
Pulmonary hypertension (PASP ≥ 50 mmHg)	46 (13.3)	25 (7.4)	15 (4.5)	6 (1.9)	<0.001	*0.002*	*0.545*	*0.085*	0.398
Significant tricuspid regurgitation	48 (13.9)	48 (14.2)	38 (11.5)	29 (9.3)	0.055	*0.818*	*0.102*	*0.284*	0.509
NYHA III–IV	245 (71.0)	-	12 (4.2)	10 (3.2)	<0.001	*-*	*-*	*0.962*	0.564

Values are expressed as mean ± standard deviation or median (25th–75th percentiles) if continuous or n (%) if categorical. # The overall *p*-value tests whether there is a change over time in the echocardiographic findings; * The *p*-value for interaction tests whether the profile over time is different for none/mild vs. mod/severe TR. LVEF: left ventricular ejection fraction; LVEDD: left ventricle end diastolic dimension; RV: right ventricle; LVESD: left ventricle end systolic dimension; PASP: pulmonary artery systolic pressure.

**Table 5:** Echocardiographic findings at different time points separately by significant and low-grade TR (subgroup analysis). In no case is the interaction of time and group significant (no difference in time profile of low-grade and significant TR; see Table 4).

					Change over Time	Post Hoc Comparisons		
	Pre-Operative	Discharge	30 Days	1 Year Follow-Up	Overall *p*-Value	Discharge vs. Preoperative	Overall *p*-Value	Discharge vs. Preoperative
Significant Tricuspid regurgitation - Low-grade (0–1+) - Significant (2–4+)	0 (0%) 48 (100%)	16 (5%) 33 (69%)	13 (5%) 25 (56%)	10 (4%) 30 (45%)	0.573 0.027		0.573 0.027	
LVEF (%) - Low-grade TR group - Significant TR group	53.9 ± 12.5 50.3 ± 11.9	54.4 ± 11.3 51.9 ± 12.7	55.5 ± 10.4 52.7 ± 11.8	55.7 ± 9.6 54.2 ± 11.2	0.005 0.040	*0.235* *0.080*	0.005 0.040	*0.235* *0.080*
Aortic peak gradient (mmHg) - Low-grade TR group - Significant TR group	71.8 ± 22.7 68.8 ± 28.2	15.5 ± 7.2 16.1 ± 8.7	16.2 ± 8.2 16.4 ± 11.7	16.4 ± 10.7 17.3 ± 14.9	<0.001 <0.001	*<0.001* *<0.001*	<0.001 <0.001	*<0.001* *<0.001*
Aortic mean gradient (mmHg) - Low-grade TR group - Significant TR group	44.8 ± 14.7 43.9 ± 19.0	8.5 ± 4.0 8.2 ± 4.7	8.8 ± 4.6 9.1 ± 6.7	9.1 ± 6.8 9.4 ± 8.4	<0.001 <90.001	*<0.001* *<0.001*	<0.001 <90.001	*<0.001* *<0.001*
LV mass (g) - Low-grade TR group - Significant TR group	214 ± 66 231 ± 102	209 ± 61 220 ± 80	200 ± 63 241 ± 95	192 ± 57 206 ± 80	0.011 0.267	*0.256* *0.357*	0.011 0.267	*0.256* *0.357*
Indexed LV mass (g/m2) - Low-grade TR group - Significant TR group	122 ± 33 135 ± 43	122 ± 43 127 ± 40	114 ± 34 130 ± 44	108 ± 30 123 ± 35	<0.001 0.299	*0.899* *0.182*	<0.001 0.299	*0.899* *0.182*
LVEDD (mm) - Low-grade TR group - Significant TR group	47.0 ± 7.7 46.9 ± 9.9	46.7 ± 7.9 47.3 ± 8.0	48.0 ± 7.9 46.2 ± 10.4	46.3 ± 7.0 48.0 ± 11.3	0.109 0.847	*0.564* *0.691*	0.109 0.847	*0.564* *0.691*
LVESD (mm) - Low-grade TR group - Significant TR group	33.5 ± 15.3 35.5 ± 12.2	32.5 ± 15.8 32.3 ± 10.1	31.9 ± 7.2 33.6 ± 9.7	31.1 ± 6.8 34.5 ± 12.1	0.101 0.056	*0.115* *0.012*	0.101 0.056	*0.115* *0.012*
Impaired RV function (RVEF < 40%) - Low-grade TR group - Significant TR group	9 (3.1%) 10 (21.3%)	7 (2.5%) 10 (23.3%)	1 (0.5%) 5 (15.1%)	0 (0%) 3 (10.3%)	0.209 0.295	*0.557* *0.649*	0.209 0.295	*0.557* *0.649*
Pulmonary hypertension (PASP ≥ 50 mmHg) - Low-grade TR group - Significant TR group	27 (9.3%) 19 (40.4%)	14 (4.9%) 11 (24.4%)	9 (4.5%) 6 (18.2%)	5 (3.0%) 1 (3.4%)	0.009 0.004	*0.018* *0.061*	0.009 0.004	*0.018* *0.061*
NYHA III-IV - Low-grade TR group - Significant TR group	207 (70%) 38 (79%)	NA NA	8 (3%) 4 (10%)	7 (3%) 3 (9%)	<0.001 <0.001	*NA* *NA*	<0.001 <0.001	*NA* *NA*

Foot notes as in Table 4; vs. preop.

## Data Availability

The dataset generated and analysed during the current study is available from the corresponding author upon reasonable request.

## List of Abbreviations

CI	confidence intervals
CT-scan	computed tomography scan
HR	hazard ratios
IQR	interquartile range
LV	left ventricle
LVEF	left ventricular ejection fraction
MR	mitral regurgitation
NYHA	New York Heart Association
PCI	percutaneous coronary intervention
PM	pacemaker
SD	standard deviation
STS	Society of Thoracic Surgeons
TAVR	transcatheter aortic valve replacement
TR	tricuspid regurgitation
